# Characterisation of a transcriptome to find sequence differences between two differentially migrating subspecies of the willow warbler *Phylloscopus trochilus*

**DOI:** 10.1186/1471-2164-14-330

**Published:** 2013-05-14

**Authors:** Max Lundberg, John Boss, Björn Canbäck, Miriam Liedvogel, Keith W Larson, Mats Grahn, Susanne Åkesson, Staffan Bensch, Anthony Wright

**Affiliations:** 1 Department of Biology, Lund University, Ecology Building, Lund, SE 22362, Sweden; 2 Department of Laboratory Medicine, Clinical Research Center, Karolinska Institute, Karolinska University Hospital, Huddinge, SE 14186, Sweden; 3 Södertörn University, School of Life Sciences, Huddinge, SE 141 89, Sweden

**Keywords:** 454 Transcriptome sequencing, Genetics of migration, Phylloscopus

## Abstract

**Background:**

Animal migration requires adaptations in morphological, physiological and behavioural traits. Several of these traits have been shown to possess a strong heritable component in birds, but little is known about their genetic architecture. Here we used 454 sequencing of brain-derived transcriptomes from two differentially migrating subspecies of the willow warbler *Phylloscopus trochilus* to detect genes potentially underlying traits associated with migration.

**Results:**

The transcriptome sequencing resulted in 1.8 million reads following filtering steps. Most of the reads (84%) were successfully mapped to the genome of the zebra finch *Taeniopygia gutatta*. The mapped reads were situated within at least 12,101 predicted zebra finch genes, with the greatest sequencing depth in exons. Reads that were mapped to intergenic regions were generally located close to predicted genes and possibly located in uncharacterized untranslated regions (UTRs). Out of 85,000 single nucleotide polymorphisms (SNPs) with a minimum sequencing depth of eight reads from each of two subspecies-specific pools, only 55 showed high differentiation, confirming previous studies showing that most of the genetic variation is shared between the subspecies. Validation of a subset of the most highly differentiated SNPs using Sanger sequencing demonstrated that several of them also were differentiated between an independent set of individuals of each subspecies. These SNPs were clustered in two chromosome regions that are likely to be influenced by divergent selection between the subspecies and that could potentially be associated with adaptations to their different migratory strategies.

**Conclusions:**

Our study represents the first large-scale sequencing analysis aiming at detecting genes underlying migratory phenotypes in birds and provides new candidates for genes potentially involved in migration.

## Background

Animal migration requires an integrated set of physiological, morphological and behavioural adaptations [1-3]. Some of these adaptations are directly associated with migration such as organ plasticity, fat deposition and orientation ability. Other adaptations also influence animals during the non-migratory period. For example, in birds, the timing of migration has to be coordinated with the timing of breeding and moult. Several of the traits involved in migration have the potential of evolving rapidly. New migratory behaviours have been established over a few decades in European blackcaps *Sylvia atricapilla*[4] and in North American house finches *Carpodacus mexicanus*[5]. In the house finch, migratory birds have developed significantly longer wings compared to resident birds [6]. Similarly, the novel migratory behaviour observed in the blackcap, which differs in its directional and distance component from the supposedly ancestral migratory behaviour, has been paralleled by morphological changes [7].

Quantitative genetic studies of blackcap populations have shown a strong heritable basis of several components of migratory behaviour, such as timing, duration and direction [8-10]. However, despite their often strong heritable basis, only a handful of studies have explored the genetic architecture of traits associated with migration. For example, Zhu *et al.*[11] found 40 microarray probes differentially expressed between migrating and reproductive summer monarch butterflies *Danaus plexippus*. Using a candidate gene approach a length polymorphism in the 3′ untranslated region (UTR) of the *ADCYAP1* gene that was found to explain a small but significant part of the variation in migratory activity within and between different European populations of blackcaps [12]. Increased knowledge of the genetic composition of migratory traits would allow for comparative studies across different species and provide new insights into the evolution of migration. The slow progress of connecting genes to a migratory phenotype could at least partly be attributed to the fact that none of the traditional genetic model species exhibit any distinct migratory behaviour [13]. The huge amount of data that now can be obtained from non-model species using next-generation sequencing is therefore likely to accelerate the field, with the potential of exploring how sequence polymorphisms, gene expression and epigenetic processes influence migratory phenotypes.

In this study we attempt to identify genes potentially associated with migratory traits by using next-generation sequencing of a brain-derived transcriptome from two subspecies of a small passerine, the willow warbler *Phylloscopus trochilus*. In Scandinavia, *P. t. trochilus* breeds in the south and *P. t. acredula* in the north, and a hybrid zone is found in the central part of the region [14,15]. Analyses of ringing recoveries and stable isotopes in feathers moulted in wintering areas in Africa have shown that the subspecies exhibit different migratory behaviour. Southern birds migrate southwest in autumn to wintering grounds in western Africa, whereas northern birds migrate south to southeast and winter in Eastern and Southern Africa [16-18]. Previous genetic studies of mitochondrial DNA, several microsatellites and thousands of amplified length polymorphism (AFLP) loci have shown that the two subspecies could be regarded as a single panmictic population [14,15,19]. Due to this very low genomic background differentiation, loci showing large allele frequency differences between the subspecies are likely to be either themselves under divergent selection or linked to genes under divergent selection [20-25]. Given an otherwise small phenotypic differentiation, it is reasonable to assume that a majority of divergently selected genes between the two subspecies are associated with adaptations to the different migratory strategies.

With our data set we specifically wanted to investigate sequence variation in coding genes and determine how this variation is distributed between the subspecies. The sequence data provides a genome-wide set of markers and allows for detection of differences in protein-coding regions that could give rise to phenotypic differences. We used normalized cDNA libraries in order to increase the sequencing depth of low abundance transcripts and to maximize the number of sequenced genes. The normalization in combination with a pooling of samples has the consequence that expression differences could not be reliably quantified from this data set. Expression differences, which are likely to be important for differences in migratory phenotypes, are instead investigated in another study using microarray expression profiling of the same samples (Boss *et al.* in prep). Here we describe the use of genomic resources of a closely related bird species, the zebra finch *Taeniopygia guttata*, to assemble and annotate reads, and demonstrate the efficiency of this approach in detecting sequence variation in a non-model species.

## Results

### Sequencing and assembly

Two runs of 454 sequencing produced 1,312,317 and 1,197,626 raw reads, respectively (Table 1), with a similar contribution from each of the subspecies. As expected from the improvement of the sequencing technology, the average read length was higher for the second run. Following linker and vector removal, 1,804,166 reads (71.9%) comprising 565 Mb remained for assembly. Of these reads, 1,515,232 (84.0%) were mapped to the zebra finch genome. Together these covered 69.8 Mb (5.7%) of the genome. The alignments were distributed on all chromosomes with a strong positive correlation (*R* = 0.89) between the number of reads per chromosome and chromosome length. A small number of reads (20,835) were defined as chimeric by the mapping software and had two separate alignments included in the output, which in the vast majority of cases were situated on different chromosomes. The mapping quality (MQ) of reads ranged from 0 to 40, with a mean value of 32.9. MQ is the phred-scaled probability (−10 log_10_*P*) that a read has been incorrectly aligned in the genome, with for example a value of 40 corresponding to a probability of 0.0001 [26]. In order to determine whether excluding alignments with lower MQ values would influence the results, sequencing depth and SNP statistics were also calculated for 1,223,938 reads with a MQ ≥ 30. This filtered set of reads was overlapping with fewer positions in the zebra finch genome and generally showed reduced sequencing depth as compared to the full set of reads. However, it did not qualitatively differ with respect to number of genes, relative sequencing depth among different types of features or the distribution of differentiation indices (Additional file 1: Table S1).

**Table 1:** Read statistics for the two different 454 runs

	**Run 1**	**Run 2**	**Total**
**Raw reads**			
Number of reads	1,312,317	1,197,626	2,509,943
Total length (Mbp)	408.0	430.8	838.8
Mean read length (bp)	310.9	359.7	334.2
Min read length (bp)	40	40	40
Max read length (bp)	698	837	837
**Filtered reads**			
Number of reads	955,919	848,247	1,804,166
Total length (Mbp)	278.2	286.7	565.0
Mean read length (bp)	294.5	338.0	313.1
Min read length (bp)	46	40	40
Max read length (bp)	552	837	837

### Annotation

**Figure 1:**
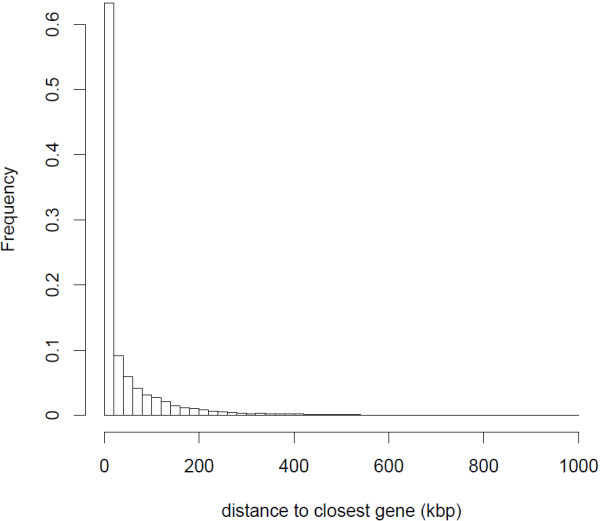
**Frequency distribution of distances to the closest gene feature for 32,351,947 aligned positions in intergenic regions of the zebra finch genome.** Excluded are 4,912,443 positions located on chromosomes with unplaced contigs (e.g. chr3_random and chrUn).

The annotation resulted in 32,525,078 (46.6%) of the aligned nucleotide positions overlapping with one or more gene feature in the zebra finch genome. These overlapping gene features originate from 12,101 unique genes when excluding positions where features from multiple genes were overlapping. Including positions in multiple overlapping genes, the number of unique genes was 12,975. Within genes, more aligned positions were located in introns than in exons, but the mean sequencing depth was higher in exons than in introns (Table 2). Of positions in intergenic regions, most were located relatively close to a gene feature (Figure 1). The distance ranged from 1–986,072 bp with a mean and a median of 45,741 and 7,175 bp, respectively. Alignments in intergenic positions were sometimes located close to genes that had no reads mapping within them. For example, if the annotated interval of genes in the zebra finch genome was expanded by 2,000 bp upstream and downstream then alignments of willow warbler reads overlapped with an additional 1,703 annotated genes.

**Table 2:** Sequencing depth and SNP statistics for different features

**Feature**	**Number of bp**	**Mean sequencing depth (range)**	**Number of SNPs**	**SNP density (SNPs/1000 bp)**
**All**	69,789,646	6.4 (1–3,148)	162,503	10.7
**Intergenic**	37,264,390	5.9 (1–3,148)	102,217	12.3
**Genic**	32,525,078	7.0 (1–1,738)	60,286	8.8
Exon	9,872,566	15.6 (1–1,738)	36,254	8.5
Intron	21,373,444	3.0 (1–561)	20,604	9.3

SNPs refer to high-quality SNPs (minor allele frequency ≥ 0.05 and the minor allele present in at least three reads). SNP density was calculated by dividing the number of SNPs with the number of positions with a minimum sequencing depth of six reads, which is the lowest depth in which a SNP can be detected using the filtering requirements.

### SNP extraction and validation

**Figure 2:**
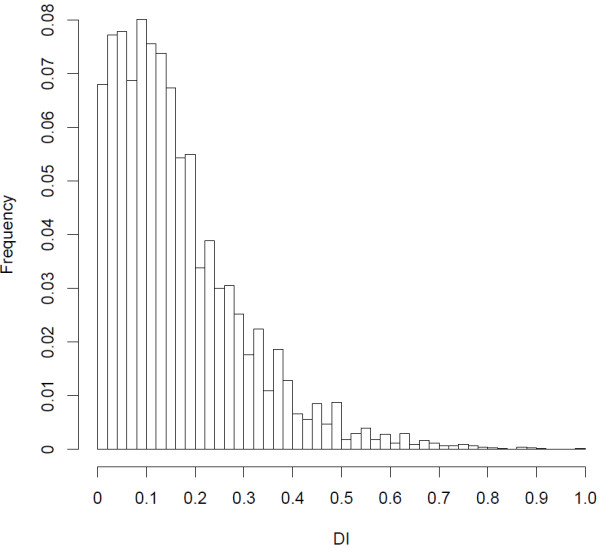
Frequency distribution of differentiation indices (DI) of 84,847 high-quality SNPs with a minimum sequencing depth of eight reads/subspecies pool.

**Figure 3:**
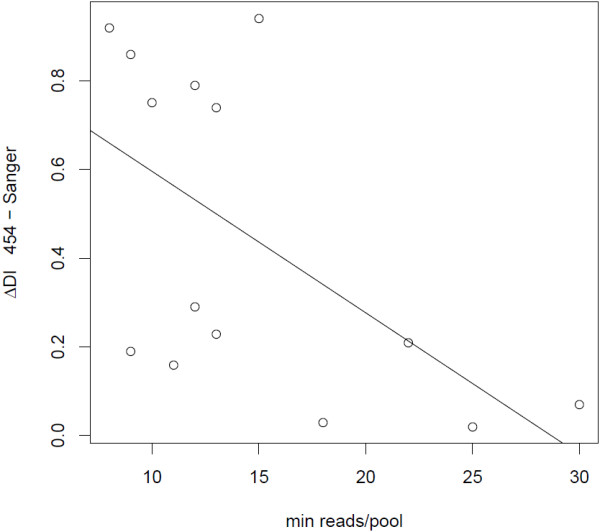
**Relationship between minimum number of reads per pool and difference in differentiation index (DI) between the 454 and Sanger validation set for 14 SNPs.** The line refers to a least-squares regression line.

The SNP extraction pipeline detected 1,557,265 raw SNPs in the willow warbler reads mapped to the zebra finch genome. With a requirement of a minor allele frequency of at least 0.05, and a presence of the minor allele in at least three reads, the total number of high-quality SNPs was 162,503. The density of SNPs was quite similar in different feature categories, with the highest value in intergenic regions and the lowest in exons (Table 2). Genetic differentiation between the pools was quantified for each SNP using a differentiation index (DI), which obtains a maximum value of 1.0 when two different alleles are fixed in each of the pools (see Methods for a more detailed description). The average DI between the subspecies was consistently low using different thresholds of minimum number of reads from each pool (Figure 2, Table 3). However, only a very small proportion of the SNPs were highly differentiated (DI ≥ 0.9). For example, with a requirement of eight reads from each of the pools, 55 out of 84,847 SNPs were highly differentiated (Figure 2, Table 3, Additional file 2: Table S2). Of 14 highly differentiated SNPs validated with Sanger Sequencing of genomic DNA in an independent set of eight samples from each of the subspecies, all but one SNP associated with the *ADCYAP1R1* gene were successfully genotyped in all the samples (Table 4). SNPs with the highest number of reads/pool generally had the smallest difference in DI between the 454 transcriptome and validation data sets (*R* = −0.58, *p* = 0.03; Figure 3). Eight of the SNPs possessed large allele frequency differences (DI > 0.6) between the subspecies in the validation set and were clustered in two regions on chromosome 1 and chromosome 5 in the zebra finch genome (Table 4). These SNPs also showed similar levels of differentiation between eight Lithuanian willow warblers, which belong to the northern subspecies (*acredula*), and willow warblers from southern Sweden in the validation set (Table 4). This suggests that the identified SNPs represent subspecies-specific differences rather than adaptations to the different environmental conditions found in northern and southern Sweden.

**Table 3:** Number of SNPs and distribution of differentiation indices (DI) as a function of minimum number of reads from each subspecies pool

**Min reads/pool**	**Number of SNPs**	**Mean DI**	**Highly differentiated SNPs**
8	84,847	0.17	55
9	77,656	0.16	41
10	71,451	0.15	25
11	65,811	0.15	18
12	60,771	0.14	15
13	56,303	0.14	11
14	52,065	0.13	8
15	48,421	0.13	6
16	45,137	0.13	5
17	42,001	0.12	4
18	39,126	0.12	4
19	36,333	0.12	3
20	33,772	0.12	3
21	31,402	0.11	3
22	29,226	0.11	3
23	27,164	0.11	2
24	25,092	0.11	2
25	23,301	0.11	2
26	21,663	0.11	1
27	20,120	0.11	1
28	18,877	0.11	1
29	17,689	0.11	1
30	16,723	0.10	1

**Table 4:** Highly differentiated SNPs validated with Sanger sequencing

**Gene name**	**Position in zebra finch genome**	**Min reads/pool**	**DI 454**	**DI Sanger S Sweden N Sweden/ S Sweden Lithuania**
RNF6*	Chr1: 48,509,942	13	0.92	0.69/0.56
RB1*	Chr1: 56,558,186	9	1.00	0.81/0.50
ESD*	Chr1: 57,059,422	12	0.92	0.63/0.38
ESD*	Chr1: 57,059,472	11	0.91	0.75/0.43
*XPOT*	Chr1A: 32,952,256	10	0.94	0.19
*ADCYAP1R1*	Chr2: 3,348,189	8	0.92	0.00
*RNMT*	Chr2:101,951,215	13	0.93	0.19
*RNMT*	Chr2:101,951,219	12	0.92	0.13
*BAI3*	Chr3: 86,028,013	9	0.92	0.06
ENSTGUG00000005084*	Chr5: 3,850,065	18	0.91	0.88/0.88
FADS3*	Chr5: 6,375,371	30	0.95	0.88/0.94
FADS3*	Chr5: 6,375,565	25	0.96	0.94/0.94
FADS3*	Chr5: 6,375,583	22	0.96	0.75/0.69
*SORD*	Chr10: 3,477,546	15	0.94	0.00

Of the 14 candidate SNPs identified by the transcriptome analyses, eight ^(*)^ showed high to moderate differentiation (DI > 0.6) between southern (N = 8) and northern Swedish samples (N = 8) when independently validated by Sanger sequencing. These eight SNPs also showed elevated DIs between southern Swedish and Lithuanian samples (N = 8). The SNP in *ADCYAP1R1* could only be successfully genotyped in four southern and five northern Swedish samples in the validation set.

## Discussion

Next-generation sequencing provides new opportunities for studying the genomics of non-model organisms [27]. For recently diverged taxa connected by gene flow, such as the two subspecies of the willow warbler, genomic differences are expected to be few but informative about adaptive divergence [28,29]. On the other hand, detecting these differences could require a dense set of markers. The sequencing of a willow warbler transcriptome has vastly increased the genomic resources available for this species. Previous studies on the same populations have used a small number of coding genes or microsatellites [14,15,30] or anonymous AFLPs [19] to study genetic divergence between the subspecies. With the use of the zebra finch genome for annotation, this data set provides a large amount of sequence data that could be associated with particular genes and gene features. As such, it will be a useful resource for future research by highlighting potentially interesting genes or genomic regions and by aiding the design of primers surrounding sequence polymorphisms.

In this study, the vast majority of reads (84%) were matched to the zebra finch genome, but the applicability of cross-species genome mapping is dependent on the similarity between the genomes and is not expected to work equally well among different non-model species. The specific karyotype of the willow warbler has not been determined and it is not known how the arrangement of genes within and between chromosomes differs between the willow warbler and zebra finch genome. Within the order Passeriformes (passerines), to which both the willow warbler and zebra finch belong, a remarkable conservation of genome structure has been reported, with both gene order and gene content on chromosomes being largely unchanged even between distantly related species [31-33]. It therefore seems plausible that the zebra finch genome is a good model for the genome of the willow warbler. However, it is not necessarily identical, since chromosomal re-arrangements have been observed between different species of passerines [31,32] and a neo-sex chromosome is suggested to have arisen in a subgroup of passerines presumably including the willow warbler [34]. On the other hand, these types of large-scale differences in genome organization have little impact on positions within or in the immediate vicinity of genes and do not therefore cause major differences in annotation at the gene level. Another possibility is that some coding genes found in the willow warbler genome are not present in the zebra finch genome. Of particular concern are genes underlying traits associated with migration, since the zebra finch does not show behavioural adaptations (e.g. migratory restlessness and hyperphagia) that are observed among long-distance avian migrants [13]. However, migratory behaviour has been characterized as a threshold trait in which a non-migratory phenotype is switched into a migratory phenotype when the combined effects of multiple genes reach a threshold [35,36]. As a threshold trait, it is possible for variation responsible for migratory behaviour to be maintained in a population consisting of only resident individuals [37]. It is therefore likely that migration genes are also found in the zebra finch genome even if they do not manifest themselves into a distinct migratory phenotype in contemporary zebra finch populations.

As expected from the enrichment of mature mRNA in the sample preparation steps, the greatest sequencing depth was observed in exons (Table 2). Unexpectedly, however, alignments in intronic features were common and, compared to alignments in exons, spread out over more positions in total in the zebra finch genome. This could be explained by that the enrichment step might not have been perfect and included unspliced mRNA [38]. More than half of the positions in the zebra finch genome with aligned willow warbler reads were situated in intergenic regions, but the sequencing depth was lower than within annotated genes. As found in a transcriptome sequencing study of the great tit *Parus major*[38], most of the intergenic positions were located close to predicted genes in the zebra finch genome (Figure 1). A possible explanation is that a majority of the positions could be situated in uncharacterized parts of UTRs. More distantly situated alignments could potentially be situated in genes that have not been annotated in the zebra finch genome. Positions in putative UTRs are of importance because they add more sequence data to genes and thus make it possible to identify more sequence polymorphisms. Additionally, in some cases they constitute the only aligned positions associated with a certain gene.

A large number of SNPs were detected in the sequence data, which were located on all but a handful of small chromosomes. The difference in SNP density observed across features is likely to be associated with their different evolutionary constraints. For example, exonic positions had a lower density of SNPs than what is found in both intronic and intergenic positions (Table 2). Overall, the SNP density is lower than found in a previous sequencing study of the subspecies [39]. A likely explanation is that the sequencing depth in general is too low to include many of the rarer alleles in the pools. This explanation is supported when restricting analyses to SNPs with a greater sequencing depth. For example, if only positions with a sequencing depth of at least 60 reads are included, the SNP density is nearly doubled and more similar to previous estimates.

Due to the construction of the cDNA library it is impossible to trace the sequencing efficiency of each individual and this could bias the genetic differentiation estimate (DI) for each SNP. In order to get a more unbiased estimate, we filtered SNPs by their minimum sequencing depth in each of the pools. Assuming that all or most individuals in each pool have similar expression levels of transcripts, and that these pooled transcripts are randomly sequenced, increasing the sequencing depth makes it more probable that more individuals are represented among the sequence reads. However, larger expression differences between individuals would result in a higher probability for transcripts from certain individuals to be sequenced. In this case, filtering by sequencing depth may not necessarily provide a more unbiased estimate. Nevertheless, the general distribution of DIs of SNPs between the subspecies pools (Figure 2, Table 3) agrees with the low background differentiation and few genetic differences previously reported between the subspecies [14,15,19]. For example, with a minimum requirement of eight reads from each subspecies pool, only 55 out of 84,847 SNPs had a DI ≥ 0.9 (Figure 2, Table 3). Of these, four are located within a chromosome region that was previously shown to be highly differentiated between northern and southern willow warblers in Scandinavia [39]. Eight of the 14 remaining highly differentiated SNPs that were validated were also differentiated between the subspecies in an independent set of individuals originating from southern and northern Sweden (Table 4). The difference in DI between the 454 data and the validation data was generally smaller with an increased sequencing depth in both of the pools in the 454 data set (Figure 3). This suggests that filtering SNPs by the sequencing depth provides an efficient way of accounting for sequencing bias of individuals in the pools. The validation set included a particularly interesting SNP that is situated close to the *ADCYAP1R1* gene. This gene encodes a membrane receptor that binds to the product of the *ADCYAP1* gene [40], which has been shown to explain some of the migratory behaviour observed within and between European blackcap populations [12]. Even though not all individuals in the validation set could be successfully genotyped for this particular SNP, there was no difference in allele frequency between the genotyped samples.

The identified genetic differences between southern and northern Swedish willow warblers could also reflect adaptations to different environments. The only obvious large environmental and ecological contrast within the sampling area is between the Scandinavian mountains and the rest of Scandinavia. In Sweden, only the northern subspecies occurs in the mountains and some adaptations to this drastically different environment are expected. Indeed, two alleles of an earlier identified genetic marker show a distribution that is strongly associated with the different environments [19]. To address this question, we also genotyped eight individuals caught in Lithuania for SNPs that showed a moderate to high differentiation between southern and northern Swedish samples in the validation set. Willow warblers in Lithuania belong to the northern subspecies (*acredula*) and express the same migratory behavior as northern birds in Scandinavia. The environment, however, is more similar to what is found at the same latitude in Southern Sweden. If the highly differentiated SNPs identified in this study were associated with adaptations to the different environments of southern and northern Sweden, we would expect the Lithuanian birds to have genotypes much more similar to birds in Southern Sweden. In contrast, we observed comparable levels of differentiation for SNPs between southern Swedish and Lithuanian samples as between southern and northern Swedish samples. This corroborates the hypothesis that the identified genetic differences are associated with the subspecies in general and are potentially linked to adaptations involved in their different migratory strategies.

The majority of the highly differentiated SNPs, including most of the SNPs verified to be differentiated in the validation set, are not located in the protein coding part of genes, but in intergenic regions (Additional file 2: Table S2). Although some of these SNPs themselves might be directly under divergent selection, they are more likely to be differentiated because they are in linkage disequilibrium (LD) to divergently selected variation in the closest gene or even in genes further away on the same chromosome. The requirement of a minimum sequencing depth in each of the pools used for estimating differentiation reduces both the number of SNPs within genes and the number of genes containing SNPs. For example, the almost 85,000 identified SNPs with a minimum sequencing depth of eight reads in each of the pools are located in at least 2,469 predicted genes. Including positions overlapping multiple genes and positions 2000 bp upstream and downstream of the genes, the total number of genes is increased to 3,642, which still is only a fraction of the number of genes covered by the total set of sequence data. Hence it is possible that many sequence variants are missed because of insufficient sequencing depth. In addition, LD has previously been shown to extend over several Mb in the willow warbler. Using the zebra finch genome as a basis for gene order, Lundberg *et al.*[39] identified a region on chromosome 3 that was highly differentiated between willow warblers in the Scandinavian mountains and the rest of Fennoscandia. The chromosome region is significantly more differentiated than an estimated genomic background level for at least a 2.5 Mb interval in the zebra finch genome that contains at least 24 coding genes. In the present study, SNPs that also were highly differentiated in the validation set clustered in regions comprising 8.5 Mb on chromosome 1 and 2.5 Mb on chromosome 5 in the zebra finch genome (Table 4). Since the distances are based on positions in zebra finch genome, they should only be regarded as rough approximations of those found in the willow warbler genome and in reality these SNPs might be located closer or further away from each other. Potentially large divergent chromosome regions could be formed if selection is reducing gene flow between chromosomes possessing alleles that are favourable in different environments, [24,28,41]. Reduced gene flow could also be facilitated by an inversion, which could maintain favourable allele combinations despite gene flow [23,42,43].

With the present data set it is possible that most of the large to moderate-sized divergent chromosome regions between the subspecies could be identified, but in order to detect more genomically localized differences, a denser set of markers would be required. This has been shown in a recent study of three-spined sticklebacks *Gasterosteus aculeatus*[44], in which full-genome re-sequencing identified a number of more genomically localized signals of divergent selection that had not been detected in a previous genome scan employing 45,000 SNPs originating from Restriction-site Associated DNA (RAD) tags [45]. Another limitation of the present study design is that the low number of individuals in each of the pools only allow for detection of SNPs that are highly differentiated between the subspecies. Smaller allele frequency differences could be expected if a locus is more loosely linked to a gene under selection, if weaker divergent selection is acting on the trait [23] or if the genetic architecture of a trait is primarily composed of many loci with small effects [46].

Future work will aim at validating the remainder of the highly differentiated SNPs in other individuals from the same populations of willow warblers. If these SNPs also are found highly differentiated in a validation set, their allelic distribution will be investigated over a larger geographical scale to determine how well it follows the distribution of the subspecies. Putative divergent chromosome regions will be more finely mapped to get a better approximation of their size and gene content. When the differentiated regions have been properly delimited, efforts should be focused on identifying the actual targets of selection. This process will be aided by the integration with data derived from a microarray expression profiling study performed on the same set of samples (Boss *et al*. in prep). A particularly interesting analysis would be to compare the genomic position and the functional annotation of differentially expressed genes between migrating individuals of each subspecies with to those of genes found within the differentiated chromosome regions.

## Conclusions

This study represents the first large-scale sequencing analysis in birds that attempts to explore the genetic architecture of traits involved in migration. The allele frequency distribution of SNPs identified in reads mapped to the zebra finch genome confirmed previous studies showing that most of the genetic variation is shared between the subspecies. A major finding was the discovery of two chromosome regions that are likely to be under divergent selection between the two subspecies and that could be involved in adaptations to their different migratory strategies. Genetic variation within these chromosome regions will be integrated with microarray expression profiling data from the same samples in order to find networks of potential genes encoding migratory traits that could be investigated in other study systems.

## Methods

### Sample preparation

Eight males of each subspecies were caught during time of breeding in Southern Sweden (ssp. *trochilus*, Krankesjön, 55°43′N, 13°25′E, 8–19 May 2008) and in Northern Sweden (ssp. *acredula*, Tångböle (N = 4), 63°22′N, 12°36′E, 30 May 2008; Anjan (N = 4), 63°43′N, 12°32′E, 31 May 2008). Eight individuals of each subspecies were also caught during migration in Southern Sweden (ssp. *trochilus*, Krankesjön, 15–16 August 2008) and in Northern Sweden (ssp. *acredula*, Skeppshamn, 62°23′N, 17°44′E, 19 August 2008). Given the high natal dispersal of the species [14], males caught at the same site during breeding are likely to be unrelated and thus represent independent samples of the subspecies originating from a larger area. Willow warblers caught during migration are presumably even more unrelated because of the funnelling of migrating birds that is typically observed at stop-over sites. Thus the included set of individuals is likely to be a representative sample of each of the subspecies in Sweden. Capturing was performed using mist nets and song playback with ethical permission from the Swedish Environmental Protection Agency and the Swedish Bird Ringing Center (ringing licence 555). Once caught, birds were decapitated and had their brain immediately put in RNAlater™ RNA stabilization agent (Qiagen, Hilden, Germany). The collection was performed with an ethical permission from Malmö/Lund Committee for Animal Experiment Ethics (no. M22-05). In the lab, brains were homogenized in 1 ml of QIA-zol Lysis Reagent (Qiagen, Hilden, Germany) per 100 mg tissue using a Tissuelyser (Qiagen, Hilden, Germany). Total RNA was extracted using RNeasy Lipid Tissue Mini Kit (Qiagen, Hilden, Germany) following the manufacturer’s instructions. The quality of the samples was checked on a formaldehyde agarose gel and using an Ultraspec 300 spectrophotometer (Pharmacia Biotech, Uppsala, Sweden) to measure 260/280 absorption (all values were between 1.95 and 2.05).

### cDNA library preparations

Of the 32 samples in total, eight from each subspecies were selected for construction of cDNA libraries. These were evenly represented by samples collected during time of breeding and migration, and constitute a subset of samples that have been used in a microarray expression profiling study (Boss *et al.* in prep.). Two μg of total RNA from each individual were pooled together to form subspecies-specific pools. Messenger RNA (mRNA) was isolated from 5 μg of pooled total RNA by exonuclease digestion followed by lithium chloride precipitation using the mRNA-only Eukaryotic mRNA Isolation Kit (Epicentre, Madison, WI, USA). Synthesis and amplification of cDNA was performed with the Mint-Universal cDNA synthesis Kit (Evrogen, Moscow, Russia) with 1 μg of mRNA used for first-strand cDNA synthesis. 800 ng of amplified cDNA was used to create a normalized library with the Trimmer kit (Evrogen, Moscow, Russia).

### 454 sequencing

454 libraries were constructed according to the manufacturer’s standard protocols (Roche/454 life sciences, Branford, CT 06405, USA). Nebulization was used to randomly shear cDNA into appropriately sized sequencing fragments of 400–900 bp. This method is optimized for shearing high molecular weight genomic DNA, but is expected to lead to a sequencing bias towards the middle and the end of a transcript [47]. In order to obtain a more even sequencing depth of transcripts, cDNA was prior to nebulization ligated into linear concatemers using sfi linkers. Concatemers were on average 10 kb and contained eight to nine cDNAs. Each of the subspecies-specific cDNA libraries were sequenced on half a picotitre plate using the 454 GS FLX Titanium technology. Sequencing was performed in two runs, first at AGOWA (Berlin, Germany, May 2010) and then at the Department of Biology, Lund University (Lund, Sweden, January 2011). Because of the improvement of the sequencing technology, the second run was expected to give on average longer reads. Combining the runs, each subspecies had been sequenced on an entire picotitre plate. The sequence data has been deposited in the NCBI sequence read archive (SRA) under accession number SRA056327.

### Genome mapping

Prior to genome mapping, sfi linkers used in concatemerization were removed from reads using a perl script provided by AGOWA (Berlin, Germany). In order to remove potentially contaminated sequences, standalone blast (blastn) was used to match reads against the pDNR-lib vector (Clontech, Mountainview, CA, USA) used in the cDNA library construction. Reads obtaining a hit with an E-value lower than 1E-5 to the vector were discarded from downstream analyses. The zebra finch genome assembly version 3.2.4 was downloaded from the UCSC genome website http://genome.ucsc.edu/ and was used as a reference genome for mapping. The assembly contains 33 chromosomes (including mitochondrial DNA) and three linkage groups. It also includes 33 sequences (e.g. chr1_random) that are comprised of contigs that are associated with particular chromosomes but which have not been successfully ordered within them, and one sequence (chrUn) derived from contigs that have not been unambiguously mapped to any of the chromosomes or linkage groups. Trimmed reads were mapped to the zebra finch genome using GMAP version 2012-01-11 [48] with a cross-species alignment flag. For each read, the best alignment to the genome was kept in the output, with the exception of chimeric reads, for which multiple alignments were retained. Reads from the different subspecies pools were mapped separately and had their output stored in a Sequence Alignment/Map (SAM) file format.

### Calculation of sequencing depth and SNP extraction

SAM files were converted into Binary Alignment/Map (BAM) format and sorted using the view and sort function in Samtools version 0.1.18 [49]. A pileup file with per basepair information from the alignments was generated with the mpileup function in Samtools using both of the subspecies-specific BAM files and the zebra finch genome as input files. The raw pileup file included positions in gaps of spliced alignments, presumably in introns, that did not contain any aligned nucleotides. A customized perl script was used to filter away these positions and for the remainder count the number of each nucleotide in each of the pools. Any insertions or deletions were ignored, but ambiguous nucleotides (Ns) were included when calculating the sequencing depth for each position. Single nucleotide polymorphisms (SNPs) were defined as positions where at least two different nucleotides (alleles) were present. For a position to be categorized as a high-quality SNP, the minor allele had to show a frequency of at least 0.05 and be present in at least three reads. For positions with more than two alleles, the minor allele referred to the second most common nucleotide.

For each SNP, a differentiation index (DI) was calculated between the southern and the northern pool as follows. The proportions of each of the four nucleotides in each of the pools were first calculated and then subtracted between the pools. DI represents the largest absolute difference in nucleotide proportions and ranges from 1, which occurs when reads from each subspecies pools are fixed for different alleles, and 0 when there is no difference in allele frequencies between the two subspecies pools. Highly differentiated SNPs were arbitrarily defined as SNPs with a DI ≥ 0.9. This is likely to be a conservative threshold to highlight SNPs that have a large allele frequency difference between the subspecies pools and that could be under divergent selection. Due to the construction of the cDNA library, reads could only be assigned to any of the subspecies pools and not to individuals within them. With a decreasing number of reads in each pool, the probability increases that not all of the individuals are represented. To account for this, SNPs were filtered based on the minimum number of reads from any of the pools. This threshold was varied in order to determine its effect on the general distribution of DI and the number of highly differentiated SNPs between pools. A subset of highly differentiated SNPs (N = 14), which included some with the highest number of reads in each of the pools, was validated in eight other individuals of each of the subspecies sampled in Sweden (Additional file 2: Table S2 and Additional file 3). Genetic differences between southern and northern willow warblers in Sweden could, in principle, reflect adaptations to the different environments found between the Scandinavian mountains in the north and the rest of Sweden, rather than being subspecies-specific. Indeed, the two alleles of a previously identified genetic marker AFLP-ww1 are strongly associated with these contrasting environments [19]. SNPs showing high to moderate DI in the validation set of Swedish samples were therefore also genotyped in eight Lithuanian samples (Additional file 3: Table S4) and had a DI calculated between them and the southern Swedish samples in the validation set. The Lithuanian samples belong to the northern subspecies (*acredula*), but are as willow warblers in southern Sweden (*trochilus*), homozygous for the southern allele of AFLP-ww1 [15]. If the SNPs were associated with AFLP-ww1 and the different environmental conditions between the north and south of Sweden, a much smaller DI would be expected between the southern Swedish and the Lithuanian samples than between southern and northern Swedish samples in the validation set.

### Annotation

Positional information of zebra finch genome features was downloaded as a General Transfer Format (GTF) file from the Ensembl website http://www.ensembl.org. Information on exons was extracted from the file and converted into a Browser Extensible Data (BED) file using a customized perl script. For each transcript, positions of introns were inferred from genomic intervals between consecutive exons. The final file contained 315,974 features from 18,618 genes and 19,334 transcripts. No features were available for chromosome 16. Positional information extracted from the pileup file of the willow warbler alignments was also converted into BED file format and compared to the zebra finch feature BED file using Bedtools version 2.15.0 [50]. For comparison, the closest command was used with a -d flag, which reports the distance to the closest feature if no overlapping feature is found. Based on the annotations, a nucleotide position was classified as either intergenic or genic. The genic category was further divided into an intronic and exonic category. If a position was overlapping with both introns and exons from different transcripts of the same gene, or overlapping with features from multiple genes, it was not included in the intron or exon category. For each of the different categories, the total number of nucleotide positions, the sequencing depth and the number of SNPs were calculated. Based on the results of previous transcriptome studies using the zebra finch genome [38,51], a fraction of reads were expected to be located in intergenic regions. Following the approach by Santure *et al.*[38], the distribution of distances from intergenic positions to the closest feature was investigated to determine whether most of the positions were close to predicted genes and therefore could be situated in putatively uncharacterised UTRs. Positions located in any of the chromosomes containing unplaced contigs (e.g. chr1_random and chrUn) were excluded from this analysis. All statistical analyses and graphics were performed with R version 2.14.1 [52].

## Competing interests

The authors declare that they have no competing interests.

## Authors’ contributions

MLu performed genome mapping, annotation, various downstream analyses and drafted the manuscript. JB and BC assisted in several initial analyses of the transcriptomic data. SB, MG and AW conceived the study. All authors provided comments and approved the final manuscript.

## Supplementary Material

Additional file 1: Table S1 Comparison of sequencing depth, number of aligned positions and SNP statistics between the full set of aligned reads and the subset of aligned reads with a mapping quality (MQ) ≥30. Click here for file

Additional file 2: Table S2 Highly differentiated SNPs (DI ≥0.9) with a minimum of eight reads from each of the sequencing pools. Click here for file

Additional file 3 **Additional information on validation procedure.** Description of primer design, amplification and Sanger sequencing of sequences containing highly differentiated SNPs. **Table S3** provides information on primers used for amplification and sequencing. **Table S4** provides information on the independent set of samples that have been used for validation. Click here for file
