# Supplementary material for: Characterisation of a transcriptome to find sequence differences between two differentially migrating subspecies of the willow warbler Phylloscopus trochilus
Source: BMC Genomics. 2013 May 14;14:330. doi: 10.1186/1471-2164-14-330 (PMC3660185; doi:10.1186/1471-2164-14-330)
Supplement: Additional file 3 — Additional information on validation procedure. Description of primer design, amplification and Sanger sequencing of sequences containing highly differentiated SNPs. Table S3 provides information on primers used for amplification and sequencing. Table S4 provides information on the independent set of samples that have been used for validation. [file 1471-2164-14-330-S3.pdf]

Validation of highly differentiated SNPs

Subspecies alignments in BAM format were concatenated into a single BAM file using the cat function in Samtools [1]. From this alignment, a willow warbler consensus sequence spanning a few hundred bp around each SNP was copied from Samtools tview and imported into Geneious 5.5.3 [2]. Within the same genomic intervals the corresponding zebra finch sequence was also retrieved. Primers (Additional file 3: Table S3) spanning the SNP were designed using Primer 3 [3] implemented in Geneious or manually if the algorithm could not detect any suitable pair. Eight samples representing each of the subspecies from Southern and Northern Sweden (Additional file 3: Table S4), respectively, were amplified using a touchdown PCR consisting of 12 cycles with 45 s of elongation and an initial annealing temperature of 63°C, which was decreasing with increments of 0.5° C/cycle, and followed by 27 cycles of 57°C annealing temperature and 45 s of elongation. PCRs were performed in reaction of 25 µl containing 1.5 mM $MgCl_2$, 0.125 mM of each dNTP, 0.4 µM of primer, 0.5 units of Taq Polymerase (AmpliTaq®, Applied Biosystems, Branchburg, NJ, USA) and 10 ng of DNA template. Following quality checking on a 2 % agarose gel, PCR products were precipitated using 11 µl of Ammonium Acetate and 37.5 µl of 95 % ethanol. Sanger sequencing was performed using BigDye Terminator v.1.1 cycle sequencing kit (Applied Biosystems, Austin, TX, USA) on an ABI 3130 prism robot (Applied Biosystems, Foster City, CA, USA). The resulting sequencing files were visualized in Geneious and, following trimming of low-quality parts, aligned to the consensus 454-derived willow warbler sequence using MUSCLE [4] with a maximum of 10 iterations and manual adjustments if necessary. Heterozygous positions were scored manually. Sequences that are at least 200 bp long have been deposited in GenBank [GenBank: KC344860 - KC344958, KC517117-KC517140].

1. Li H, Handsaker B, Wysoker A, Fennell T, Ruan J, Homer N, Marth G, Abecasis G, Durbin R, Proc GPD: **The Sequence Alignment/Map format and SAMtools**. *Bioinformatics* 2009, **25**(16):2078-2079.

2. Drummond A, Ashton B, Buxton S, Cheung M, Cooper A, Duran C, Field M, Heled J, Kearse M, Markowitz S *et al*: **Geneious v5.4, Available from http://www.geneious.com**. 2011.

3. Rozen S, Skaletsky H: **Primer3 on the WWW for general users and for biologist programmers**. *Methods Mol Biol* 2000, **132**:365-386.

4. Edgar RC: **MUSCLE: multiple sequence alignment with high accuracy and high throughput**. *Nucleic Acids Res* 2004, **32**(5):1792-1797.

Table S3 Primers used for validation

Gene	Forward primer 5'-3'	Reverse primer 5'-3'	Amplicon size (bp)
ADCYAP1R1	TGGCTCCAGCCAAGAGMAGA	GCCGTSCTAGMGCTCCCTT	493
BAI3	AAGTCACTGAACTATTCAGAGGCC	TACAGTCTGCAGTGAGCCAGG	215
ESD	ACTCTCCCTTCTCCCTCCC	TGTTACCAGCTACACTGTGCCA	347
FADS3	CACCGAGCCTCTTCCTGCC	AGCACCTTGCTGATTMTGTGGGA	516
ENSTGUG00000005084	AGCAATGCRGAGTCATTTCAGCCT	CCTGGGTGCGCATTCACYGT	565
RB1	AGAGCTTACAGTCAGTGCACCC	GCTGATCCTAGAGTACTTACAGTAG	277
RNF6	ACCCCAGAACTGGCTGCCGA	GCCCATCCTTCGACTTGCCC	290
RNMT	TGCAGGCTTGAGCACAGCACA	CAACCGTGAAACTTCACATACTGGA	537
SORD	CAAGCGGATCAACGTCAAGCC	CCGACTGTGCAAACCATCATCTCT	306
XPOT	CCGAACCAGCAAGGCTTTCAG	TCAGAGGCAGAGGGAATGAACG	207

Table S4 Samples used for validation

Sample ID	Locality	Coordinates
96A/05	Stensoffa, Southern Sweden	55° 41' 42" N, 13° 26' 50" E
96A/13	Stensoffa, Southern Sweden	55° 41' 42" N, 13° 26' 50" E
97A/03	Haganäs, Southern Sweden	56° 15' 0" N, 14° 39' 60" E
97A/05	Haganäs, Southern Sweden	56° 15' 0" N, 14° 39' 60" E
97B/01	Växjö, Southern Sweden	56° 50' 0" N, 14° 43' 60" E
97B/10	Växjö, Southern Sweden	56° 50' 0" N, 14° 43' 60" E
97E/05	Tåkern, Southern Sweden	58° 19' 0" N, 14° 48' 60" E
97E/06	Tåkern, Southern Sweden	58° 19' 0" N, 14° 48' 60" E
01K/01	Tornehamn, Northern Sweden	68° 26' 0" N, 18° 34' 60" E
01K/02	Tornehamn, Northern Sweden	68° 26' 0" N, 18° 34' 60" E
01L/19	Stordalen, Northern Sweden	68° 19' 60" N, 19° 6' 0" E
01L/20	Stordalen, Northern Sweden	68° 19' 60" N, 19° 6' 0" E
01M/05	Kaisepakte, Northern Sweden	68° 16' 60" N, 19° 18' 60" E
01M/06	Kaisepakte, Northern Sweden	68° 16' 60" N, 19° 18' 60" E
97N/13	Gällivare, Northern Sweden	67° 13' 0" N, 20° 48' 0" E
97N/14	Gällivare, Northern Sweden	67° 13' 0" N, 20° 48' 0" E
2D/02	Voke, Lithuania	54° 35' 0" N, 25° 10' 0" E
2D/03	Voke, Lithuania	54° 35' 0" N, 25° 10' 0" E
2D/05	Voke, Lithuania	54° 35' 0" N, 25° 10' 0" E
2D/08	Voke, Lithuania	54° 35' 0" N, 25° 10' 0" E
2D/09	Voke, Lithuania	54° 35' 0" N, 25° 10' 0" E
2D/18	Voke, Lithuania	54° 35' 0" N, 25° 10' 0" E
2D/19	Voke, Lithuania	54° 35' 0" N, 25° 10' 0" E
2D/20	Voke, Lithuania	54° 35' 0" N, 25° 10' 0" E
